# A genomic approach highlights common and diverse effects and determinants of susceptibility on the yeast *Saccharomyces cerevisiae *exposed to distinct antimicrobial peptides

**DOI:** 10.1186/1471-2180-10-289

**Published:** 2010-11-15

**Authors:** Belén López-García, Mónica Gandía, Alberto Muñoz, Lourdes Carmona, Jose F Marcos

**Affiliations:** 1 Departamento de Ciencia de los Alimentos, Instituto de Agroquímica y Tecnología de Alimentos (IATA), CSIC, Apartado de Correos 73, Burjassot, 46100 Valencia, Spain; 2 Departamento de Genética Molecular de Plantas, Centre for Research in Agricultural Genomics (CRAG) CSIC-IRTA-UAB, Barcelona, Spain; 3 Fungal Cell Biology Group, Institute of Cell Biology (ICB), University of Edinburgh, Rutherford Building, Edinburgh EH9 3JH, UK

## Abstract

**Background:**

The mechanism of action of antimicrobial peptides (AMP) was initially correlated with peptide membrane permeation properties. However, recent evidences indicate that action of a number of AMP is more complex and involves specific interactions at cell envelopes or with intracellular targets. In this study, a genomic approach was undertaken on the model yeast *Saccharomyces cerevisiae *to characterize the antifungal effect of two unrelated AMP.

**Results:**

Two differentiated peptides were used: the synthetic cell-penetrating PAF26 and the natural cytolytic melittin. Transcriptomic analyses demonstrated distinctive gene expression changes for each peptide. Quantitative RT-PCR confirmed differential expression of selected genes. Gene Ontology (GO) annotation of differential gene lists showed that the unique significant terms shared by treatment with both peptides were related to the cell wall (CW). Assays with mutants lacking CW-related genes including those of MAPK signaling pathways revealed genes having influence on sensitivity to peptides. Fluorescence microscopy and flow cytometry demonstrated PAF26 interaction with cells and internalization that correlated with cell killing in sensitive CW-defective mutants such as Δ*ecm33 *or Δ*ssd1*. GO annotation also showed differential responses between peptides, which included ribosomal biogenesis, *ARG *genes from the metabolism of amino groups (specifically induced by PAF26), or the reaction to unfolded protein stress. Susceptibility of deletion mutants confirmed the involvement of these processes. Specifically, mutants lacking *ARG *genes from the metabolism of arginine pathway were markedly more resistant to PAF26 and had a functional CW. In the deletant in the arginosuccinate synthetase (*ARG1*) gene, PAF26 interaction occurred normally, thus uncoupling peptide interaction from cell killing. The previously described involvement of the glycosphingolipid gene *IPT1 *was extended to the peptides studied here.

**Conclusions:**

Reinforcement of CW is a general response common after exposure to distinct AMP, and likely contributes to shield cells from peptide interaction. However, a weakened CW is not necessarily indicative of a higher sensitivity to AMP. Additional processes modulate susceptibility to specific peptides, exemplified in the involvement of the metabolism of amino groups in the case of PAF26. The relevance of the response to unfolded protein stress or the sphingolipid biosynthesis, previously reported for other unrelated AMP, was also independently confirmed.

## Background

Antimicrobial peptides (AMP) and peptide-related molecules are widespread in nature in organisms all along the phylogenetic scale, and are considered part of an ancestral innate system of defence against pathogen attack or competition for nutrients [1]. They are small peptides and proteins with common properties such as direct antimicrobial activity, abundance of cationic and hydrophobic residues, amphipathic conformations and diverse structures. Synthetic AMP have also been either designed *de novo *on the basis of these properties or identified by means of combinatorial and non-biased approaches. AMP show great potential as alternatives to face the decreasing efficacy of conventional antibiotics in clinic [2,3], new tools in plant protection [4,5], or novel food preservatives [6,7].

In contrast with the hundreds of peptides endowed with antimicrobial activity that are currently known, only a minor proportion of them have been studied in detail in relation to their mechanism of action. Detailed knowledge of mode of action is critical to sustain the potential application of AMP. It was initially considered that microbial killing was a primary consequence of the *in vitro *membrane disturbing properties shared by many cationic and amphipathic AMP. Nevertheless, today it is established for a number of peptides that there are also non-lytic modes of action that might involve specific interactions at cell envelopes and/or with intracellular targets, even among peptides known as potentially membrane-disrupting [8-12]. Significant examples include: the binding of either the peptidic lantibiotic nisin [13,14] or the amphipathic fungal defensin plectasin [15] to the bacterial peptidoglycan precursor Lipid II; the requirement of plant defensins for the presence of distinct classes of membrane glycolipids [16-18]; the interaction of different AMP with heat shock related proteins [19-21]; or the induction of DNA damage and apoptosis [22-24]. Also, cell penetrating properties are being discovered among peptides previously known as antimicrobials and, reversibly, some penetrating-like peptides show antimicrobial potency [25].

Genome-wide techniques and transcriptional profiles have contributed to the characterization of AMP mechanisms [15]. The underlying assumption is that these studies will provide non-biased clues to the mode of action and specificity determinants of compounds (peptides), which will subsequently help in the design of new generations of improved antimicrobials, in terms of inhibitory potency and selectivity. Such analyses might also highlight novel targets for antimicrobials. Moreover, expression profiling is considered as a fingerprint to find common and distinct responses that could aid in the design of combined therapies of unrelated compounds, to which AMP might contribute. However, this type of studies are still scarce in the case of AMP, with only a few examples in bacteria [26-29] and fungi, mostly yeast [30-33]. Transcriptome profiling has been used to characterize the response of the model yeast *Saccharomyces cerevisiae *to distinct antifungals [34-39], including selected AMP [30,33].

In this study we aim to compare at a genomic scale the effects onto *S. cerevisiae *of two AMP with distinctive properties. Melittin is an α-helical membrane active peptide identified from honeybee venom that is recognized as a model pore-forming peptide for the study of peptide interaction with lipid bilayers and cell permeating properties [40]. On the other hand, PAF26 is a short *de novo*-designed hexapeptide [41], which shares sequence similarity with other AMP from natural [42] or synthetic origin [43,44]. It has activity against plant pathogenic fungi as well as several microorganisms of clinical relevance, including the yeast *Candida *and several dermatophytic fungi [45]. PAF26 at low micromolar (sub-inhibitory) concentrations has been recently shown to have cell penetrating properties in the mycelium and conidia of the filamentous plant pathogen *Penicillium digitatum *[46] and the model fungus *Neurospora crassa *(A. Muñoz and N. Read, unpublished observations). Contrary to melittin, PAF26 is less active against bacteria and is not haemolytic under assay conditions in which other peptides including melittin are [45].

We combined global analyses of transcriptomic changes upon exposure of *S. cerevisiae *to sub-lethal concentrations of either PAF26 or melittin with sensitivity tests of strains lacking genes identified by the transcriptomic data. Our results both reinforce and extend similar studies undertaken previously with two unrelated α-helical AMP [33], and reveal that PAF26 and melittin have common but also distinctive effects on yeast.

## Results

### Antimicrobial activity of peptides PAF26 and melittin against *S. cerevisiae*

**Figure 1:**
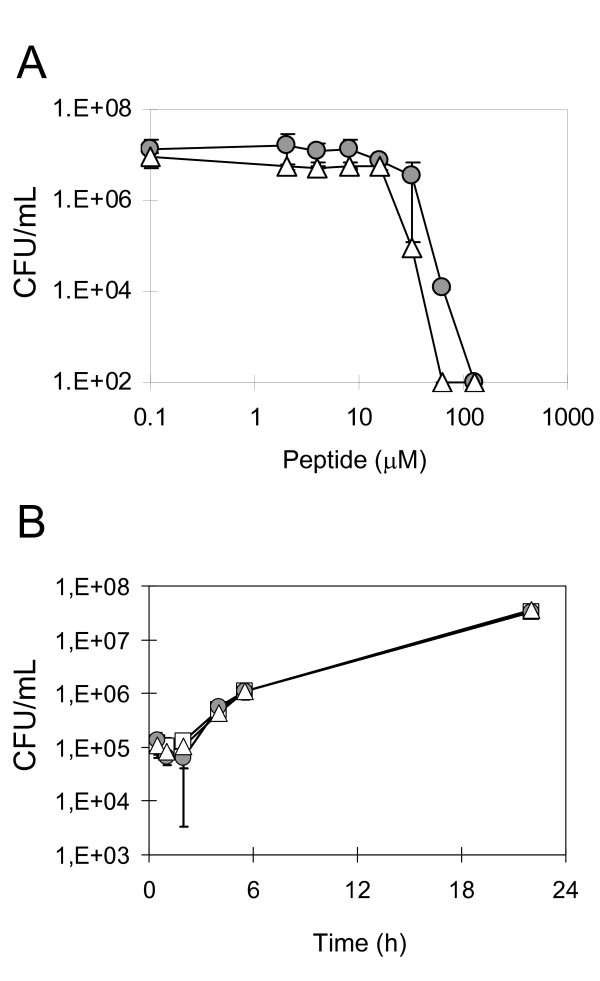
**Antifungal activity of peptides PAF26 and melittin to *S. cerevisiae *FY1679**. **(A) **Dose response curve of cell killing activity. Cells were exposed to different concentrations of peptides for 24 h. Cell survival (measured as CFU/mL) was determined by dilution and plating. **(B) **Time course of cell population growth was followed in the presence of 5 μM of peptide. No significant differences were found between each of the peptides and the control treatment. In both (A) and (B) panels, grey circles and white triangles indicate PAF26 and melittin samples, respectively; in (B), white squares show controls in the absence of peptide.

PAF26 and the pore-forming peptide melittin inhibited yeast growth [41], as was confirmed herein with strain FY1679 (Figure 1A and Additional File 1) in experiments that show a slight 2-fold higher potency of melittin. Dose-response experiments with additional strains of yeast with distinct genetic backgrounds and at two temperatures of incubation confirmed the activity of both peptides and also indicated a differential sensitivity of strains (Additional File 1). Thus, BGW7a, which has a weakened CW and is also sensitive to an increase in growth temperature and to plant antimicrobial proteins [47], was markedly more sensitive than other strains to melittin and, to a minor extent, PAF26. On the other hand, strain RAY3A [48] had a susceptibility to peptide killing similar to strains FY1679 and BY4741.

### Global transcriptome response of *S. cerevisiae *to PAF26 and melittin

In order to gain knowledge and compare the antifungal effect of PAF26 and melittin we carried out the characterization of the transcriptome of *S. cerevisiae *after exposure to these peptides. The global transcriptome response to peptides was undertaken by treating *S. cerevisiae *FY1679 cells in the logarithmic growth phase to sub-lethal concentrations (5 μM) of either PAF26 or melittin for 3 hours. Under these assay conditions, no significant effects on growth were observed for any of the two peptides even after up to 24 hours of treatment (Figure 1B). DNA macroarrays representing more than 6,000 yeast genes were hybridized with the cDNAs from treated cells. The complete data set containing the quantification of signals has been submitted to the GEO public database http://www.ncbi.nlm.nih.gov/geo/. Annotation, processing and statistical significance of expression change for each DNA probe are shown in Additional File 2. Subsequent data analysis allowed the identification of genes with differential expression after each peptide treatment, as compared with control sample in the absence of peptide. In total, 385 genes (7.4%) of the 5,174 analyzed genes were responsive to melittin treatment while 355 genes (6.8%) of the 5,230 analyzed were differentially expressed after PAF26 treatment. Additional File 3 shows additional information on the genes with higher induction or repression upon each treatment. Some examples of the most differential genes are *ARG1 *as the gene with the highest induction specific of PAF26, *PSO2 *having the highest co-induction with both peptides, or *STE5 *and *BTN2 *as the most repressed with both peptides.

**Figure 2:**
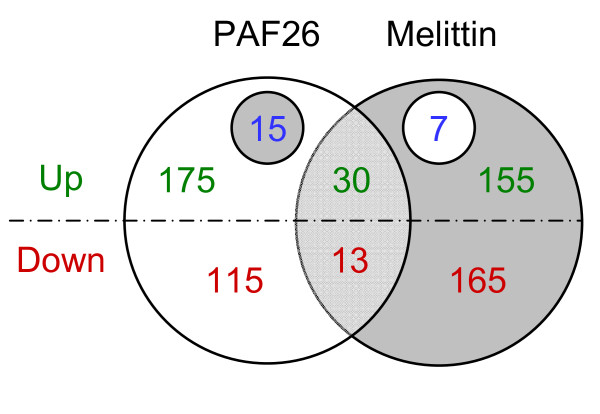
**Distribution of differentially expressed genes after peptide treatment**. A z-test for two independent conditions was conducted for each peptide treatment compared to the control treatment. Effective p-values were <3.3E-03 and <3.7E-03 for PAF26 and melittin, respectively. Diagram shows genes induced (up) or repressed (down) by peptides. The small circles on the upper part refer to 15 genes induced by PAF26 and repressed by melittin and 7 genes induced by melittin and repressed by PAF26.

Figure 2 shows the distribution of differential genes upon each treatment and emphasizes that only a minor proportion of genes co-expressed with both peptides (only 30 genes were induced and 13 genes were repressed by both peptides, see also Additional Files 3.5 and 3.6), providing an initial indication of the differential response of *S. cerevisiae *and/or to differences in the mode of action of the two peptides. Moreover, 15 genes were induced by PAF26 but repressed by melittin, while 7 were induced by melittin and repressed by PAF26. Among the former class, the two copies of the locus *CUP1 *(*CUP1_1 *and *CUP1_2*) were relevant due to their induction by PAF26 and strong repression by melittin. *CUP1 *is a copper binding metallothionein involved in resistance to toxic concentrations of copper and cadmium. Among the seven genes in the second class, we found YLR162W, which has previously been related to sensitivity of yeast to the plant antimicrobial peptide MiAMP1 [49].

We focussed on genes from MAPK signalling pathways that regulate response to environmental stresses/signals [50-52], and were also responsive to peptides. Within the HOG1 osmotic stress cascade there were several genes that responded to PAF26 but not to melittin, such as the stress-responsive transcriptional activator *MSN2 *and the phosphorelay sensing *YPD1 *that were induced, or that coding for the MAPKK PBS2p that was markedly repressed. In addition, the gene coding for the phosphatase PTC3p involved in HOG1p dephosphorylation was also markedly induced. These transcription changes related to the osmolarity HOG pathway seemed to be specific to PAF26. Within the CW growth pathway, the sensing genes *MID2 *and *RHO1 *also changed their expression upon exposure to melittin or PAF26, respectively. The only gene from these MAPK pathways that responded similarly to both peptides was the scaffold *STE5*, which in turn showed the strongest repression by both PAF26 and melittin (Additional File 3).

Only a limited number of genes coding for transcription factors were responsive to peptide treatments, and in most cases showing an induction of expression. In addition to the above mentioned *MSN2*, there were the stress-responsive *HOT1, NTH1 *and *YAP1*.

### Functional annotation analysis of the expression changes induced in response to PAF26 and melittin

Genome-scale functional annotation of the transcriptomic data was obtained by using the FatiGO tool [53], integrated in the GEPAS package http://gepas.org/[54]. This tool extracts Gene Ontology (GO) terms that are over- or under-represented in sets of differentially expressed genes, as compared with the reference sets of non-responsive genes. It also provides statistical significance corrected for multiple testing and the level of GO annotation. The complete non-redundant data set for the different combinations of treatments of our study is available in the Additional File 4. The analysis revealed significant terms among the genes that were induced and/or repressed by each peptide.

After exposure to 5 μM of PAF26, we observed up-regulation of genes involved in cell wall organization and biogenesis, belonging to the GO annotation "chitin-and beta-glucan-containing cell wall" (Additional File 4.1). Of the 14 induced genes grouped under this annotation, 6 of them were also induced after exposure to 5 μM of melittin (*plb1, tos1, pir3, pir2, dse2 *and *ecm33*). Remarkably, this cell-wall related class was the *only *significant annotation common to PAF26 and melittin treatments found in our GO analyses (Additional File 4.3).

Also significantly up-regulated by PAF26 were 5 genes belonging to the GO term "non-protein amino acid metabolic process" (Additional File 4.1), including *ARG1*, *ARG3, ARG5,6 *and *ARG7*, all involved in arginine metabolism and urea cycle KEGG pathway (http://www.kegg.com/, sce00330). All of them were significantly induced by PAF26 but were either non-induced or non-analyzed (due to threshold quality criteria) under the melittin treatment.

There were no significant GO annotations among the genes specifically up-regulated by PAF26 and that did not also respond to melittin, contrary to what occurs with the repressed genes (Additional File 4.4). Most of the genes specifically down-regulated upon exposure to PAF26 were functionally related to tricistronic rRNA processing and ribosome organization, biogenesis and maintenance (up to 82 distinct genes), small nucleolar RNA binding and also to translational initiation (Additional Files 4.1 and 4.4). The majority of these genes code for RNA binding proteins, and we have previously reported that PAF26 is capable of *in vitro *binding of tRNA from *S. cerevisiae *[46]. As an additional clue to the differential effects of both peptides, some of these categories and genes were even up-regulated by melittin (18 genes from "rRNA processing" at GO level 7, Additional File 4.2) or significantly underrepresented among the melittin-repressed genes (none of the 392 genes annotated by the biological process "RNA processing" at level 6 were down-regulated by melittin) (Additional Files 4.4 and 4.5). Moreover, there was a very significant GO annotation of "ribosome biogenesis and assembly" (adjusted *P*-value 0.00019) within the seven genes up-regulated by melittin but repressed by PAF26 (Figure 2), since six genes (i.e., *NOP1*, *CGR1*, *ALB1*, *DBP2, RPL14A*, and *UTP23*) share this term.

### Validation of gene expression changes by quantitative RT-PCR

**Figure 3:**
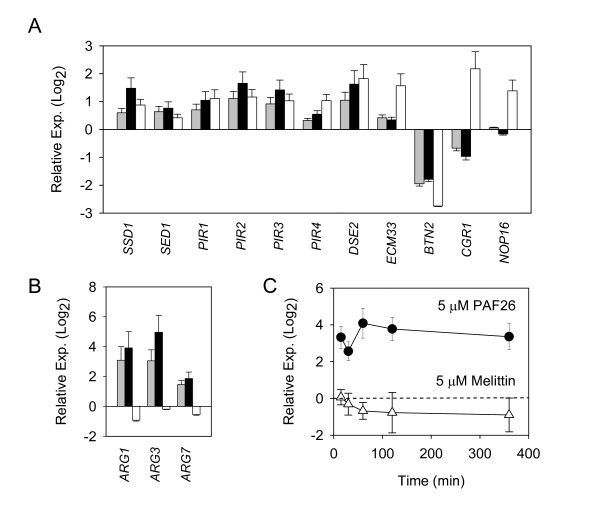
**Quantitative real time PCR analysis of gene expression changes after peptide treatment**. All the panels show the mean relative expression ± SD (y-axis) of each individual gene upon each peptide treatment as compared to the control treatment with no peptide. **(A) **and **(B) **graphs are end-point analyses of expression of the indicated genes (x-axis) after 3 h of peptide treatment; grey bars indicate 5 μM PAF26, black bars 25 μM PAF26, and white bars 5 μM melittin. Note the different expression scales in panels (A) and (B). **(C) **Graph shows time-course changes of expression of *ARG1 *following treatment with either 5 μM PAF26 or 5 μM melittin. In all the panels, the genes *ALG9*, *TAF10 *and *UBC6 *were simultaneously used as constitutive references (see Methods for details).

In order to sustain the macroarray data, 14 genes were arbitrarily selected taking into account different criteria, as the magnitude of the expression change, the differential behaviour with both peptides, or the GO annotation results; and their expression change was determined by quantitative RT-PCR (Figure 3). Thus, 8 genes (*DSE2*, *ECM33*, *PIR1*, *PIR2*, *PIR3*, *PIR4*, *SED1 *and *SSD1*) were selected as related to CW composition and organization. Representatives of genes related to ribosome biogenesis and processing were *NOP16 *and *CGR1*. Finally *ARG1*, *ARG3*, *ARG7 *and *BTN2 *were chosen because of the magnitude of their induction or repression, respectively, under PAF26 exposure. Importantly, an additional control was included in these experiments. Given that melittin was slightly more active on *S. cerevisiae *than PAF26 (Figure 1A), a five-fold higher concentration of PAF26 (25 μM) was included to rule out a peptide dose effect that might alter the interpretation of the macroarray data. Overall, this approach discards such a dose effect for a substantial number of the genes (Figure 3). The qRT-PCR results of the 14 selected genes validate the macroarray data. Notably, the differential response to peptides was confirmed for *NOP16*, *CGR1 *or the three *ARG *genes analysed (Figure 3A and 3B). The induction of *ARG1 *was around 15 times greater than control levels after exposure to PAF26 but we did not observe a significant change of expression after exposure to 5 μM of melittin (Figure 3B and Additional File 2). A similar PAF26 specific induction was confirmed for *ARG3 *and *ARG7 *(Figure 3B). The specific up-regulation of *ARG1 *was confirmed through independent experiments of treatment of *S. cerevisiae *with PAF26 or melittin, in which RNA samples were collected to quantify expression by quantitative RT-PCR in a time course experiment (Figure 3C).

### Susceptibility to PAF26 or melittin of *S. cerevisiae *deletion mutants

Considering the results described above, a set of 50 *S. cerevisiae *deletion mutants [55] were analyzed for susceptibility to PAF26 or melittin. The annotation and complete dataset of the susceptibility of mutants is found in Additional File 5. Only significant findings are discussed and shown in detail below. Deletion strains were divided into distinct groups according to their functional classification, significance or expression behaviour. Two numerous groups are related to (i) enzymes or structural proteins involved in CW composition and strengthening, and (ii) the distinct stress-sensing MAPK signalling cascades related to CW in *S. cerevisiae*. As many of these genes are related to CW composition and response, the deletion mutants were also analyzed for their sensitivity to the membrane and CW interfering agents, sodium dodecyl sulphate (SDS) and calcofluor white (CFW).

**Figure 4:**
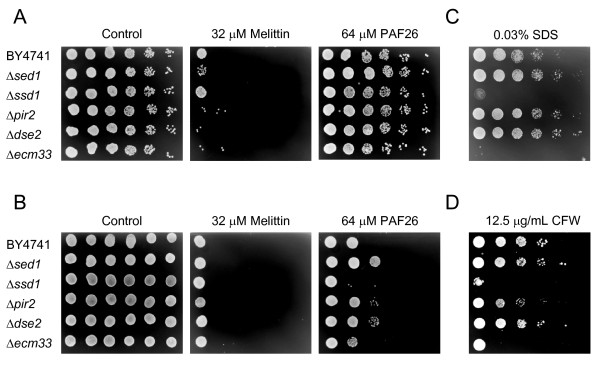
**Analysis of sensitivity to peptides and to CW disturbing compounds of *S. cerevisiae *deletion mutants in CW-related genes.** Data on sensitivity of the single gene deletion strains Δ*sed1*, Δ*ssd1*, Δ*pir2*, Δ*dse2*, Δ*ecm33*, and the corresponding parental strain BY4741 are shown. **(A) **and **(B) **show results after treatment of serial 5-fold dilutions of exponentially growing cells with each peptide for 2 hours (Panel A) or 24 hours (Panel B) and subsequent plating onto YPD peptide-free plates. **(C) **and **(D) **show growth of serial dilutions of the same deletion strains on YPD plates containing SDS (Panel C) or CFW (Panel D).

Figure 4 exemplifies our analyses in the case of structural CW proteins. From our experiments it was concluded that lethal concentrations of melittin act quicker on yeast than PAF26 under our assay conditions, since a shorter exposure to melittin (2 h) was sufficient to kill cells while a much longer time of treatment (24 h) was needed for the PAF26 effect to be noticeable (compare Figure 4A and 4B, respectively). A similar observation was found previously in the fungus *P. digitatum *[46], since melittin induced changes of mycelium quicker than PAF26. Consequently, all our experiments were conducted at least at these two exposure times and the Additional File 5 reflects the overall data obtained. A significative but minor effect on susceptibility to peptides was observed among several of the CW-related genes analyzed (i.e., only one five-fold CFU dilution difference). Despite the well-known severe lethality of Δ*ecm33*, Δ*ssd1 *and Δ*pir2 *in the presence of SDS or CFW, only a modest outcome of higher sensitivity to peptides was found (Figure 4 and Additional File 5). Function redundancy, for instance among *PIR *genes, could be partially responsible for this result. Thus, we assayed the triple mutant Δ*pir1-3 *in a different genetic background (*S. cerevisiae *RAY3A cells) [48] but did not observe a significant effect (Additional File 6), contrary to the higher sensitivity of the same strain to the antifungal plant protein osmotin [56]. In addition, the deletion of *SSD1 *in RAY3A resulted in a slight increase in sensitivity to peptides, particularly PAF26, as occurred with the corresponding BY4741 derivative. In some experiments such as the one shown in Figure 4, a slight increase in resistance was observed for Δ*sed1 *and Δ*dse2*, in response to PAF26 treatment.

Deletion strains from all the well characterized MAPK signalling pathways [50,52] were selected from at least at three points of each pathway, with an emphasis on signalling related to CW integrity and construction and osmoregulation (see Additional File 7). Some of the mutants showed a minor increase of resistance to PAF26. Several deletions tested in our experiments were, as already known, heavily affected in terms of sensitivity to SDS or CFW (i.e., Δ*pbs2*, Δ*hog1*, Δ*slt2*, or Δ*fks1*), indicating strong alterations in the CW deposition or response to stress. Remarkably, none of these and the other MAPK pathway mutants were severely affected in their sensitivity to peptides (see also Additional File 5).

**Figure 5:**
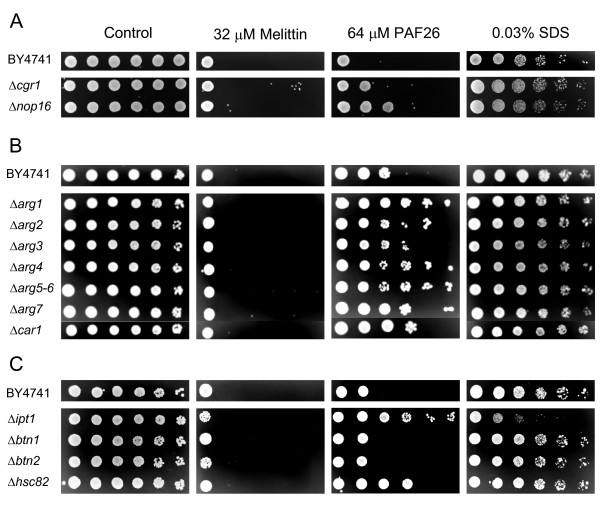
**Analysis of sensitivity to peptides and to SDS of specific *S. cerevisiae *deletion mutants**. **(A)**, **(B) **and **(C) **show results of three independent experiments, with specific genes as indicated in the figure. See the text for additional details on the selected genes. Other details as in Figure 4.

Other deletion strains were selected from the GO processes identified by functional annotation. From the three mutants tested that lack genes involved in ribosome biogenesis and RNA processing, two of them (Δ*cgr1 *and Δ*nop16*) were more resistant to PAF26 than to melittin (Figure 5A). A noticeable specific response occurred with most of the *ARG *deletants analyzed; all of them involved in the "arginine biosynthesis" and "urea cycle and metabolism of amino groups" pathways. In addition to deletants from *ARG1*, *ARG3*, *ARG5,6 *and *ARG7 *that showed a substantial specific up-regulation by PAF26, those from *ARG2*, *ARG4 *and *CAR1 *were also assayed. These seven deletants showed varying degrees of increased resistance to PAF26, which was substantial for *ARG1, ARG4 *and *ARG5,6*. Importantly, none of these strains showed phenotypes associated to CW weakening as determined by their sensitivity to SDS or CFW (Figure 5B and Additional File 5).

The *IPT1 *gene codes for the enzyme responsible of the last step in the biosynthesis of the major plasma membrane sphingolipid mannose-(inositol-P)_2_-ceramide [M(IP)_2_C] [57]. Its deletion confers resistance to other antifungals and plant antimicrobial proteins [16,58]. In our experiments, *IPT1 *expression decreased in response to melittin but not in response to PAF26. Within the same pathway, *LCB1 *encodes the enzyme of the first committed step of sphingolipid biosynthesis, and its expression was markedly repressed by PAF26 (see Additional File 3.2). The Δ*ipt1 *mutant showed a remarkable phenotype of high resistance to PAF26 combined with increased sensitivity to SDS (Figure 5C). Another mutant lacking a gene involved in ceramide synthase synthesis (i.e., *YPC1*/YBR183W) was assayed but no alteration on sensitivity to peptides was found (see details on Additional File 5).

PAF26 and related peptides are arginine-rich and penetratin-type peptides [46]. *BTN2 *codes for a protein with protein binding activity involved in amino acid transport, pH and ion homeostasis and arginine uptake [59]. It was, together with *STE5 *(see above), the gene with the highest repression common to both peptides (Figure 3 and Additional File 2). However, neither the corresponding deletion strain nor the related Δ*btn1 *[60] displayed significant differences regarding sensitivity to peptides (Figure 5C).

*HSC82 *was used as a representative of the several heat shock proteins (HSP) that are markedly repressed by PAF26 and/or melittin such as *HSP78*, *HSP12 *or *STI1 *(Additional File 3). Indeed, the response to unfolded protein stress GO term was significantly repressed upon melittin treatment (Additional File 4). *HSC82 *was repressed by PAF26, and the corresponding deletion strain was selectively more resistant to PAF26 (Figure 5C).

### Interaction of PAF26 with S. cerevisiae cells

**Figure 6:**
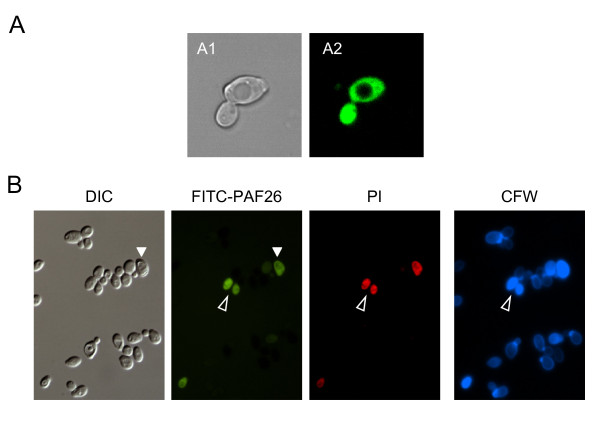
**Fluorescence microscopy of *S. cerevisiae *exposed to FITC-PAF26**. **(A) **Internalization of FITC-PAF26 into *S. cerevisiae *FY1679 demonstrated by confocal fluorescence microscopy. Cells were exposed to 30 μM FITC-PAF26 for 30 min. Bright-field (A1) and fluorescence (A2) micrographs of the same field are shown. **(B) **Interaction of FITC-PAF26 with *S. cerevisiae *BY4741 visualized by fluorescence microscopy: DIC bright field image, as well as FITC, propidium iodide (PI), and calcofluor white (CFW) signals of the same field are shown. Cells were incubated with 30 μM FITC-PAF26 at 30°C for 2 h, and then at 20°C with 2 μM PI and 25 μM CFW for 5 min. Open arrowheads indicate peptide internalization (compare location of the CW outer signal of CFW with the internal signal of PI and the FITC fluorescence resulting from FITC-PAF26). Solid arrowhead indicates the lower FITC signal in the vacuole compared to the cytosol.

We have previously reported that PAF26 is capable to interact with and be internalized by the hyphal cells of the filamentous fungus *P. digitatum *at sub-inhibitory concentrations (0.3 μM) [46]. PAF26 is markedly less active against *S. cerevisiae *than towards *P. digitatum *[41] and, accordingly, although internalization of fluorescently labeled PAF26 into *S. cerevisiae *FY1679 could be demonstrated through confocal microscopy, 100-fold higher peptide concentrations (30 μM) were required (Figure 6A).

In order to determine whether the sensitivity to PAF26 is correlated with the interaction and uptake of the peptide into *S. cerevisiae*, and also how this is associated with cell viability, we set up an assay in which cells were treated with FITC-PAF26 followed by treatment with the cell death marker propidium iodide (PI) and the CW stain CFW (Figure 6B). Approximately 5-20% of *S. cerevisiae *BY4741 were labeled by FITC-PAF26 under these assay conditions (see also below), and such labeling co-localized with that of PI. Also, staining by CFW showed strong cell wall disorganization for those non-viable cells into which peptide were located. Despite not using confocal optics as in Figure 6A, this three-fluorophore staining also supports the internalization of the peptide and confirmed that cells showing the highest peptide signal were the most permeable to PI. Our microscopy experiments also show FITC-PAF26 accumulation in the cytosol, excluded from the vacuole (Figures 6A and 6B).

**Figure 7:**
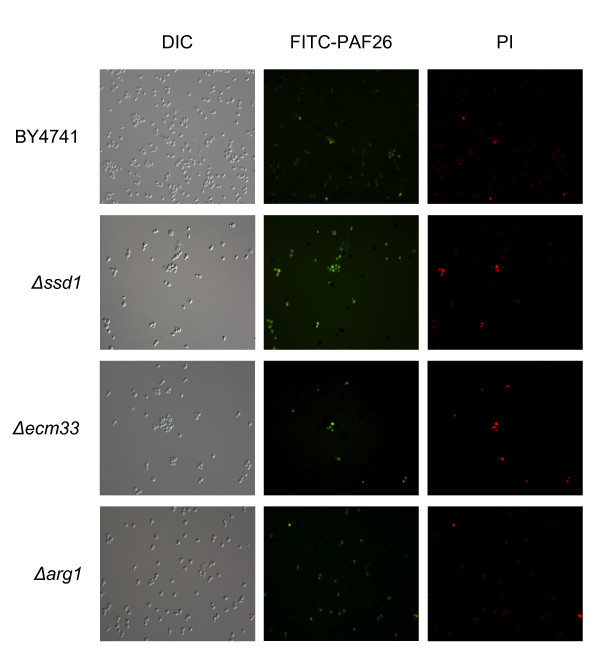
**Differential interaction of *S. cerevisiae *deletion mutants with FITC-PAF26**. Representative fluorescence micrographs of the parental BY4741 and *S. cerevisiae *deletion strains Δ*ssd1*, Δ*ecm33*, and Δ*arg1*, as indicated at the left. Optical and image acquisition settings were the same for each fluorophore and thus differences in fluorescence intensity among strains reflect real differences. Others details as in Figure 6B.

**Figure 8:**
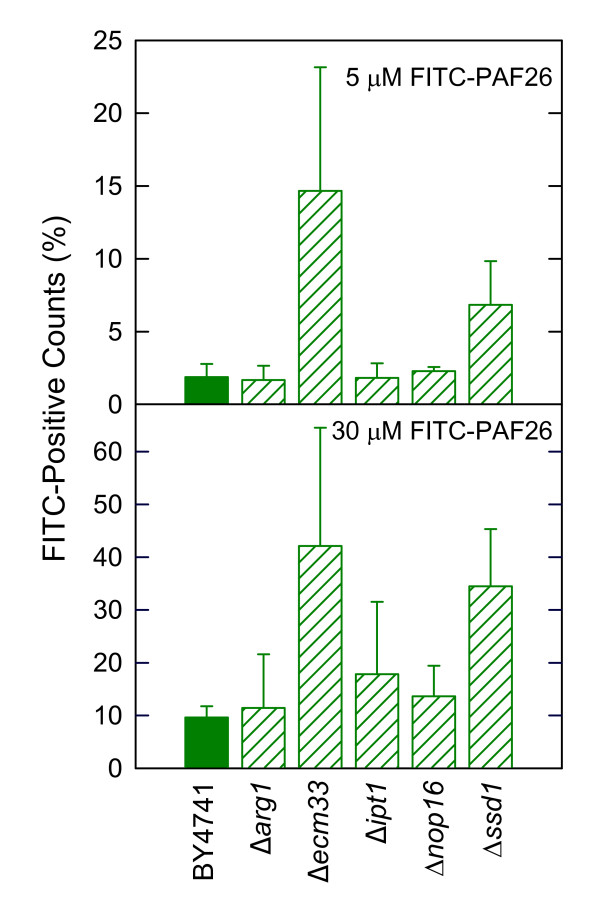
**Differential interaction of *S. cerevisiae *deletion mutants with FITC-PAF26**. Flow cytometry measurements of FITC-PAF26 binding to *S. cerevisiae *deletion mutants shown below as compared with the parental strain BY4741. Graph shows the percentage of fluorescence bound to cells after exposure of 20,000 cells to either 5 (upper panel) or 30 μM (lower panel) FITC-PAF26. Mean and SD from two replicas in each of two independent experiments are shown for each strain.

Selected deletion mutants were analyzed using this approach (Figure 7, high magnification and data on CFW staining are not shown for simplicity). Microscopical observations were validated by quantification of labeled PAF26 binding by flow cytometry (Figure 8). Deletion strains in genes involved in cell wall construction such as *SSD1 *or *ECM33 *showed a correlation with the higher sensitivity to PAF26 in that a proportion of cells higher than in the parental strain were labeled by the peptide and showed intense staining by PI. However, the resistant Δ*arg1*, Δ*nop16 *or Δ*ipt1 *mutants did not show a noticeable difference of peptide labeling as compared with the parental strain (Figure 8) and in some experiments, such as the one shown in the corresponding panel of Figure 7 (Δ*arg1*), a higher proportion of cells were labeled with the peptide. This latter result indicates that the higher resistance of these strains is not due to lack of interaction and/or internalization of the peptide.

## Discussion and Conclusions

We have carried out a functional genomic approach on yeast to gain insight into the mechanism of two AMP that presumably have different modes of antifungal killing. Analogous reports have addressed the mode of action of distinct antifungal agents [35-38,61,62], including other AMP [30,32,33]. These latter studies on AMP used inhibitory concentrations and found an array of multifactorial effects, but could not distinguish those processes primary related to peptide mechanism from those secondarily derived from cell death. Since we have observed biological changes of *P. digitatum *after exposure to sub-inhibitory (sub-micromolar) concentrations of PAF26 that include peptide internalization [46], we decided to use non-inhibitory concentrations of AMP in the gene expression experiments (5 μM, Figure 1) in an attempt to unveil primary effects of the peptides. Also, by choosing two peptides with differentiated interactions with fungal cells, we could isolate processes both common and specific of each one. The transcriptomic data demonstrates specific and statistically significant changes under these conditions that our fungicidal assays demonstrate that are involved in sensitivity to peptides.

We have combined the identification of differentially expressed genes with the use of global functional annotation tools [63]. From the wealth of available data (see Additional Files 2, 3, 4, 5), we highlight in this report the most relevant conclusions. First, our study reinforces the idea that cell permeation is not the only mechanism required to fully describe the effect of, and response to, AMP in microorganisms [8-12]. We have also shown that PAF26 and melittin have common but also differential effects on yeast. Finally, a previously overlooked observation is that a significant part of the response relies on genes of unknown function, or with poorly informative GO terms associated to them.

A remarkable example of uncharacterized genes uncovered in our study is YLR162W, the only gene not related to ribosome biogenesis among the seven induced by melittin and repressed by PAF26 (Figure 2). It is a predicted gene of unknown function that codes for a small protein with potential transmembrane domains [49]. An independent study has shown that over expression of YLR162W confers resistance to the plant antimicrobial peptide MiAMP1 in a susceptible yeast strain [49]. Strikingly, our study indicates (in a different yeast genotype) that YLR162W reacts distinctly to different AMP, and thus highlights the interest of studying its function since it might have an important and distinctive role in the response to AMP. BLAST searches do not show any homolog of this gene in known fungal sequences (data not shown).

### The role of the fungal cell wall in susceptibility to AMP

The most obvious shared response is related to reinforcement of the cell wall. Among the 43 genes that were co-expressed in the peptide treatments (Figure 2), the only GO significant annotations were related to the fungal CW (Additional File 4.3). Additional studies found altered genes involved in CW maintenance in response to other antifungal agents or CW perturbants as well [38,61,62]. Among the previous genomic studies of the response to AMP in yeast, only the one that used the esculentin 1-21 peptide highlighted CW responses at the transcriptomic level [30], while others did not [32,33]. In addition, six genes (different from those found herein) were identified whose deletions confer increased sensitivity to either dermaseptin S3 or magainin 2 [33]. Our observations sustain that the improvement of CW integrity is a common response of *S. cerevisiae *to AMP. Further support arises from the data on BWG7a strain, which has a weakened CW phenotype related to a dysfunctional *SSD1 *allele [47] that compromises viability in the presence of AMP and at higher incubation temperatures (Additional File 1). Yeast cells are capable of reinforcing their CW when subjected to stress or damage conditions [64], and our study contributes to demonstrate that this is also the case after AMP treatment. The use of BWG7a has allowed the isolation of genes that partially overcome sensitivity to the plant antimicrobial protein osmotin, such as yeast *PIR *genes or *Fusarium *homologs of *SSD1 *and *SED1 *[47,65]. On the other hand, deletion of specific CW proteins sensitize yeast to the antibacterial lantibiotic nisin [66]. Further, PAF26 induced severe mycelial growth and cell-shape defects to the fungus *P. digitatum *[46], changes that are typical for compounds affecting the cell wall.

Our assays showed only a limited number of gene deletions related to CW that have an effect on sensitivity to PAF26 or melittin. Even in these examples, the magnitude of the phenotype of the mutants (i.e., changes in sensitivity) is modest compared to that of mutants related to ribosome biogenesis, arginine metabolism, sphingolipid or HSP related genes (compare Figures 4 and 5). This holds even for genes such as the above mentioned *SSD1*, which mediates deposition of other CW proteins in *S. cerevisiae *[56]. The corresponding deletion strain has a damaged CW as confirmed by hyper-sensitivity to SDS or CFW, but comparatively only a minor increase in susceptibility to AMP as demonstrated in two genetic backgrounds (BY4741 and RAY3A, see also Additional File 6). A similar phenotype was observed in other strains such as Δ*ecm33*. Microscopy and flow cytometry studies in Δ*ssd1 *or Δ*ecm33 *showed a correlation between a higher sensitivity and an increase of PAF26 uptake of cells (Figure 7), demonstrating that CW components modulate the interaction with peptides.

Function redundancy might explain the lack of a dramatic change in the susceptibility in mutants related to CW. Therefore multiple deletions would be expected to have a higher impact and are being studied in our laboratory. However, our current data do not support this view either, as illustrated with the triple deletion of *PIR *genes in the RAY3A background (Additional File 6). Even in the case of gene deletions from MAPK signalling cascades involved in CW construction and response to stress [51], we did not find major differences in sensitivity to peptides under our assay conditions (Additional File 7). Representative examples are *STE2 *that was highly repressed by both peptides, or *SLT2*, *PBS2 *and *HOG1*, whose deletants are hypersensitive to CW interfering compounds. This result contrasts with previous data in which mutations in the HOG osmoregulatory pathway in the case of the peptide histatin [31] or the RHO1-SLT2 CW growth pathway in a plant defensin Pn-AMP1 [67] result in hypersensitivity.

Other CW-related gene deletions did not show significant differences in susceptibility to peptides and even in a limited number of examples (as Δ*sed1*) a slight higher resistance was observed. It has been described that specific gene deletions result in counteracting mechanisms to reinforce CW by enhancing levels of specific CW constituents [64]. Also, it can not be discarded that lost of specific CW proteins/components results in increased resistance due to reduced peptide binding to cell surface as shown for a dermaseptin derivative that seems to bind CW protein mannosyl phosphate residues [68]. Under this view, an attractive hypothesis that needs to be tested is that the different CW proteins identified in ours and previous studies reflect differences in the interaction of distinct AMP with the cell envelope [30,33].

### Involvement of the *IPT1 *gene in AMP sensitivity

A noteworthy mutant with a marked increased resistance to PAF26 was Δ*ipt1 *(Figure 5C). Sphingolipids are essential components of the plasma membrane in all eukaryotic cells and IPT1p catalyzes the last step in the biosynthesis of the fungal sphingolipid M(IP)_2_C. Previous works with the Δ*ipt1 *mutant showed high resistance to DmAMP1, an antifungal plant defensin, and lower peptide binding to the yeast surface than the wild-type strain [16]. The authors proposed that DmAMP1 interacts with this sphingolipid to exert the antifungal action [69]. In addition, mutants lacking the *IPT1 *gene also showed resistance to syringomycin E [58]. We found that the Δ*ipt1 *strain was resistant to PAF26 (Figure 5C) but bound FITC-PAF26 to the same extent as the parental strain (Figure 8), in contrast to what was reported in the plant defensin example. This apparent contradiction could be explained considering that the initial binding of PAF26 to the fungal cell occurs at the CW, and not at the plasma membrane. Also remarkably, Δ*ipt1 *resistance to PAF26 is coincident with extreme sensitivity to the membrane perturbing SDS (Figure 5C). This differential behaviour was unique among the strains analyzed in our study. It neatly demonstrates that although interaction is the first step in the antimicrobial mechanism of peptides, other additional susceptibility factors exist since an abnormal membrane and/or weakened CW does not always lead to higher susceptibility to PAF26 and other antimicrobial peptides/proteins. Overall, the data indicate that involvement of *IPT1 *and presence of M(IP)_2_C in the yeast plasma membrane could be a common factor for distinct AMP to exert their action onto *S. cerevisiae*.

### Intracellular effects of PAF26

An overlapping response to distinct AMP seems to be related to DNA breakdown and/or induction of apoptosis [23,24,33]. No significant annotation related to DNA damage or apoptosis was found in our GO analyses (Additional File 4). However, the gene with the highest induction (around 10-fold) by both peptides was *PSO2*, which was not identified in any of the previous studies. It is highly induced after DNA strand breaks and binds damaged DNA. On the other hand, the DNA ligase gene *DNL4 *required for non-homologous end-joining (NHEJ) and repair of dsDNA breaks is among the most repressed by both peptides. Strikingly, *LDB7 *also involved in NHEJ was the only gene repressed by two unrelated AMP [33], demonstrating that independent studies point to the same processes even though they identify distinct individual genes. We have previously shown that both PAF26 and melittin share with other cationic AMP the capacity to bind nucleic acids *in vitro *[33,46,70]. It remains to be determined to which extent this binding activity has a functional significance *in vivo*.

GO profiling demonstrated a prominent differential effect related to rRNA processing and ribosomal biogenesis, which were repressed by PAF26 but induced by melittin. A high number of genes from these annotations showed this marked differential response with extremely significant p-values (Additional File 4), including the group of seven genes induced by melittin and repressed by PAF26 (Figure 2), and was also confirmed by quantitative RT-PCR in selected genes (Figure 3A, *CGR1 *and *NOP16*). The repression behavior is shared in the response to other AMP, antimicrobial compounds and additional stress conditions [35,38,61]. mRNAs from ribosomal proteins and rRNA processing enzymes are predicted to destabilize under stress conditions [71]. It is assumed that shutdown of ribosome biogenesis and thus protein translation will free cell resources to cope with a hostile environment. However, our study opens additional questions as to the significance of the induction (rather than repression) of this response in the case of melittin, or of the increased resistance to PAF26 in some of the corresponding deletion strains such as that of the nucleolar protein NOP16 (Figure 5A).

The gene *BTN2 *has been reported to modulate arginine uptake through down-regulation of the CAN1p arginine permease [59]. Our study shows that *BTN2 *was one of the most repressed gene by both peptides (Additional File 3), suggesting that the cell is sensing the high arginine levels caused by peptide internalization and mounts an active response to deal with it. GO profiling indicated the specific involvement of the "nonprotein amino acid metabolic process" in the response to PAF26, including genes from the biosynthesis or arginine, metabolism of amino groups and urea cycle (*ARG1*, *ARG3, ARG5,6 *and *ARG7*), which were induced by PAF26 but not by melittin. *ARG1 *was the gene with the highest PAF26-specific induction identified in our macroarray study, and such strong expression change was confirmed through qRT-PCR analysis (Figure 3). *ARG1 *codes for the argininosuccinate synthase and is known to be transcriptionally repressed in the presence of arginine. Induction of these genes is indicative of attempt of metabolization of the high concentration of amino groups of cationic AMP such as PAF26. In fact, their induction could lead to accumulation of derived metabolites in the cell. Although the question of ammonium toxicity in yeast is still controversial [72], we speculate that this could be the case given the higher resistance to PAF26 of the deletion mutants assayed. In any case the high resistance to PAF26 of a number of *ARG *gene deletants confirms the involvement of these pathways in the peptide killing mechanism (Figure 5B). Importantly, susceptibility to PAF26 did not correlate with peptide interaction/internalization into cells in Δ*arg1 *(Figure 7). This mutant also combines a significant amount of peptide labelling with high resistance, confirming that peptide binding and toxicity are separate processes.

Numerous chaperone-related genes respond to PAF26 and/or melittin, and the GO term "response to unfolded protein stress" was significantly repressed by melittin (Additional File 4.2). The co-chaperone regulator of chaperone activity *STI1 *was the fifth most repressed gene by both peptides (Additional File 3.6). HSC82p and HSP82p are the two isoforms of the HSP90-like chaperone in yeast and are among the most abundant proteins in the cytosol [73]. *HSC82 *is considered to be constitutive while *HSP82 *is strongly induced by heat stress; the corresponding proteins are involved in folding of recalcitrant and denatured proteins. Contrary to *HSP82*, the *HSC82 *gene was strongly repressed by PAF26 and the deletion strain was more resistant to PAF26 killing (Figure 5C). Previous reports suggest that although nearly identical in sequence, these two isoforms are not functionally equivalent [73]. Our study provides additional data on the involvement of protein chaperones and heat shock proteins in antimicrobial peptide mode of action, which has been invoked in previous reports that include yeast and bacterial studies [9,20,21,26]. Among the eleven chaperones repressed by melittin we found *SSA2*, coding for the CW protein that together with SSA1p was shown to bind the AMP Histatin 5 and promote peptide internalization [21].

In summary, our findings help to confirm that permeation is not the unique effect of these and other AMP, and that additional (might be also overlapping) mechanisms that go beyond cell lysis are involved. The data presented support the idea that CW reinforcement and modification are part of a general fungal response to peptides with different modes of action. However, a weakened CW is not necessarily indicative of a higher sensitivity to AMP. The importance of the response to unfolded protein stress or the sphingolipid biosynthesis, previously reported for other unrelated AMP, was also confirmed independently, therefore suggesting their broad contribution to activity of antimicrobial peptides. This study has also uncovered additional processes and genes that will be further analyzed in the near future, as is the case of the involvement of the metabolism of amino groups in the case of PAF26 or the YLR162W gene.

## Methods

### Synthesis of peptides

PAF26 was purchased at >90% purity from Genscript Corporation (Piscataway, NJ, USA) and was acetylated at the N-terminus and amidated at the C-terminus. PAF26 was also synthesized labeled with fluorescein 5-isothiocyanate (FITC) by covalent modification of its N-terminus with FITC. Melittin was provided by Sigma-Aldrich (Cat nº M2272). Stock solutions of peptides were prepared in 10 mM 3-(N-morpholino)-propanesulfonic acid (MOPS) pH 7 buffer and stored at -20°C. Peptide concentrations were determined spectrophotometrically.

### *Saccharomyces cerevisiae *strains

*S. cerevisiae *strain FY1679 (MATa/MATα; ura3-52/ura3-52; trp1Δ63/TRP1; leu2Δ1/LEU2; his3Δ200/HIS3; GAL2/GAL2) was used in the macroarray assays. To test the effect of gene deletion on the activity of peptides we used the *S. cerevisiae *strains BY4741 (MATa; his3Δ1; leu2Δ0; met15Δ0; ura3Δ0) and the corresponding isogenic deletion strains from the Euroscarf public collection http://web.uni-frankfurt.de/fb15/mikro/euroscarf, as well as RAY3A (MATa; his3; leu2; ura3; trp1) and derived deletion strains [48].

### DNA macroarray experimental procedure

25 ml cultures of 10^5 ^colony forming units (CFU)/ml of *S. cerevisiae *FY1679 were grown with shaking at 30°C in 20% YPD medium (100% YPD is 1% yeast extract, 2% peptone and 2% dextrose). After 3 hours of growth, 250 μl of a 100X stock solution of each peptide were added to each yeast culture (final concentration 5 μM). The same volume of MOPS buffer was added to the control sample. Cultures were grown at 30°C with shaking for 3 additional hours. Yeast cells were collected by centrifugation and kept at -80°C until processed for RNA isolation. Three independent biological replicates were conducted for each treatment.

Total RNA was extracted from cell pellets and ethanol precipitated. Radiolabelled cDNA was obtained by reverse transcription (RT) of 20 μg of total RNA, after annealing to 3.75 μg of the anchor oligonucleotide oligo(dT)VN (Invitrogen), in the presence of 5 mM DTT, 800 μM each of dATP, dTTP and dGTP, 5 μM dCTP, 5 μl of 3000 Ci/mmol α^33^P-dCTP, 10 units RNase inhibitor (Invitrogen), and 400 units SuperScript III reverse transcriptase (Invitrogen), at 50°C for 2 h. Template RNA was removed by alkaline hydrolysis, followed by neutralization. Unincorporated nucleotides were separated from the ^33^P-labelled cDNA probe by passage through MicroSpin S-300HR columns (Amersham).

The nylon filters from the macroarray containing 6,020 yeast ORF (Laboratory of DNA chips, Universitat de València, http://scsie.uv.es/chipsdna/) with platform accession number GPL4565 at Gene Expression Omnibus (GEO) database http://www.ncbi.nlm.nih.gov/geo/, were hybridized with ^33^P-labelled cDNA probes and stripped as described [74]. A total of three different filters were used, and each biological replicate from each of the three treatments (control, 5 μM PAF26, and 5 μM melittin) was hybridized to a distinct filter. Therefore, each individual filter was subjected to three cycles of hybridization and stripping. Filters were exposed for 5-7 days to an imaging plate (BAS-MP 2040, FujiFilm), which was scanned in a phosphorimaging scanner (FLA-3000, FujiFilm).

### Analysis of the macroarray hybridizations

Quantification, normalization and statistical analysis of macroarray hybridization results were carried out with the software packages ArrayVision v8.0 and ArrayStat v1.0 (Imaging Research Inc.). The local background was defined as the mean signal intensity of an area around each block of 16 hybridized spots, and subtracted from each signal. Resulting signals were log transformed and normalized both within replicates and across treatments by the iterative median procedure. Probe replicates within a treatment were marked as outliers and removed if deviated from the mean of the replicates plus or minus two times the standard deviation. A minimum number of valid replicates of 2 was set to calculate the mean value for every probe (i.e., only 1 replicate was allowed as outlier). Each peptide treatment was compared separately against the control treatment. To identify differentially expressed probes, a z-test for two independent conditions was performed with false discovery rate (FDR) correction for multiple tests (nominal alpha value of 0.05).

The complete data set has been deposited in NCBI's Gene Expression Omnibus http://www.ncbi.nlm.nih.gov/geo/ and are accessible through GEO Series accession number GSE25279 http://www.ncbi.nlm.nih.gov/geo/query/acc.cgi?acc=GSE25279.

Lists of either induced or repressed genes upon each treatment, and the combinations of them, were generated and subjected to Gene Ontology (GO) profiling using the FatiGO tool from the GEPAS package http://gepas.org/[53,63]. Annotations were considered significant when p-value adjusted for multiple testing was lower than 0.05

### Quantitative real time PCR

Two micrograms of total RNA from each sample were treated with RNase-free DNase (Ambion), and retrotranscribed with SuperScript III reverse transcriptase (Invitrogen), essentially as described above. Real Time PCR was performed using a LightCycler 480 Real-Time PCR System (Roche Diagnostics), according to manufacturer´s protocols using the LightCycler 480 SYBR Green I Master (Roche Diagnostics), with the following thermal profile: activation step (95°C for 10 min); amplification step (40 cycles of 95°C for 10 s, 55°C for 10 s, 72°C for 10 s); melting curve program (95°C for 10 s, 60°C for 15 s, 95°C with a heating rate of 0.1°C/s); and cooling step (40°C for 30 s). Primers for the target genes *SED1*, *PIR1*, *PIR2*, *PIR3*, *PIR4*, *SSD1*, *BTN2*, *ECM33*, *CGR1*, *NOP16*, *ARG1*, *ARG3*, *ARG7*, as well as *ACT1*, *ALG9, TAF10 *and *UBC6 *as independent reference genes [75,76], were designed to an equal annealing temperature of 57°C (primer sequences are listed in Additional File 8). The quantification cycle point (Cq) for each transcript was obtained using the LightCycler 480 SW 1.5 (Roche Diagnostics). Three technical of each one of the three biological replicates were conducted. The algorithm geNorm http://medgen.ugent.be/~jvdesomp/genorm/[76] demonstrated expression stability of the three references genes *ALG9, TAF10 *and *UBC6 *under our experimental conditions. The Relative Expression Software Tool (Multiple Condition Solver REST-MCS v2) was used to determine the relative quantification of target genes normalized to the three references genes [77].

### In vitro antimicrobial activity assays

*S. cerevisiae *cells were grown to exponential phase (OD_600 _0.4-0.5) in YPD medium at 30°C with shaking. In order to quantify survival of yeast after peptide treatment, aliquots of yeast at 10^4 ^CFU/mL in 20% YPD were incubated with different concentrations of peptides for 24 h, diluted and spread onto peptide-free YPD agar plates to monitor CFU recovery. In kinetic assays, 10^5 ^CFU/mL of yeast were incubated with 5 μM of peptides in 20% YPD at 30°C for different times from 15 min to 24 h, and the CFU recovery was also quantified by spreading onto peptide-free plates.

For experiments with the different *S. cerevisiae *strains and deletion mutants, cultures were adjusted to 10^7 ^cells/ml in 20% YPD and serial 5-fold dilutions of cells were prepared and subjected separately to peptide treatment. The treatments contained 45 μl of each yeast dilution and 5 μl of a 10X stock solutions of each synthetic peptide, and were incubated in sterile 96-well microtiter plates (Nunc) at 30°C for either 2 or 24 h. Aliquots (5 μl) of each sample were dotted onto peptide-free YPD agar plates to determine viability after 2 h or 24 h of incubation. In all experiments, YPD medium contained 40 μg/ml chloramphenicol (to avoid bacterial contamination) and the agar plates were incubated at 30°C for 2 days to allow colony visualization and/or counting. In specific assays the temperature of incubation was 24°C.

Calcofluor white (CFW) (Sigma-Aldrich F3543) or sodium dodecyl sulphate (SDS) (Sigma-Aldrich L4509) plates were prepared to desired final concentrations in YPD agar medium. On these plates, aliquots (5 μl) of serial 5-fold yeast dilutions (or ten-fold dilution in the case of CFW plates) were spotted and growth was visualized after two days of incubation at 30°C.

### Fluorescence microscopy

*S. cerevisiae *cells were grown to exponential phase (OD_600 _0.4-0.5) at 30°C with shaking and the number of cells/ml was determined independently for each strain. Yeast at 10^8 ^cells/ml (final concentration) were incubated in sterile water with 30 μM FITC-labeled PAF26 for 0.5-2 hours at 30°C in the dark. After this incubation, cells were further incubated with 2 μM propidium iodide (PI) and 25 μM calcofluor white (CFW) for 5 min in order to check for viability/membrane integrity and cell wall structure, respectively. Yeast cells were washed and fluorescence was examined with an epifluorescence microscope (E90i, Nikon), with excitation/emission wavelengths of 488/510-560 nm for FITC detection, 544/612 nm for PI detection and 395/440 nm for CFW detection. Differential interference contrast (DIC) and fluorescence images were captured with ×40 and ×100 objectives using the software NIS-Elements BR v2.3 (Nikon).

In order to confirm peptide internalization, *S. cerevisiae *at 5 × 10^5 ^cells/ml were incubated in sterile water with 30 μM FITC-PAF26 in the dark, and visualized with a TCS SL confocal laser scanning microscope (Leica), with excitation at 488 nm and emission wavelengths at 510-560 nm.

### Flow cytometry

*S. cerevisiae *cells were prepared as detailed above and 2.5 × 10^7 ^cells were incubated in 100 μL of sterile water with FITC-PAF26 (5 or 30 μM) for 2 h at 30°C in the dark. After this incubation, the cell suspension was made up to 1 mL with sterile water. Analysis was performed using an EPICS XL-MCL flow cytometer (Beckman-Coulter, USA) equipped with an argon-ion laser emitting a 488 nm beam at 15 mW. An acquisition protocol was defined after measuring background fluorescence from non-treated BY4741 *S. cerevisiae *strain, and Δ*ssd1 *cells treated with 30 μM FITC-PAF26. Data (20,000 cells/sample) were analyzed with the Expo32 software included in the system acquisition.

## Authors' contributions

BLG carried out the macroarray experiment from design to hybridization; contributed to the sensitivity assays; initiated the qRT-PCR experiments; and helped to draft the manuscript. MG carried out most of the sensitivity assays of the yeast strains; helped in the analysis of the qRT-PCR data; deposited the array data at the GEO database; and contributed to draft the manuscript. AM participated in the initial conception of the approach; initiated the sensitivity assays; and performed the confocal microscopy experiments. LC completed the qRT-PCR experiments and carried out the corresponding analyses; and carried out the fluorescence microscopy and flow cytometry experiments. JFM conceived and coordinated the study; carried out the bioinformatic analysis of the macroarray data; and wrote the manuscript. All authors read and approved the final manuscript.

## Supplementary Material

Additional file 1 **Sensitivity of *S. cerevisiae *strains to peptides PAF26 and Melittin**. Sensitivity assays of *S. cerevisiae *strains RAY3A, BWG7a, FY1679, and BY4741 (10^5 ^or 10^4 ^CFU/mL) to different concentrations of peptides PAF26 and Melittin, at two different assay temperatures. Click here for file

Additional file 2 **Transcriptome analysis of *S. cerevisiae *FY1679 after exposure to peptides PAF26 and Melittin**. Excel File showing the annotation, signal intensity, processing and statistical significance of expression change for each DNA probe in the GPL4565 array. Click here for file

Additional file 3 **Representative *S. cerevisiae *genes that change expression after exposure to peptides PAF26 and Melittin**. Excel File showing lists of genes with the most significant induction/repression that are common or specific after exposure to peptides PAF26 and/or Melittin. Click here for file

Additional file 4 **Non-redundant global GO annotation analyses of *S. cerevisiae *genes differentially expressed upon peptide treatment**. Excel File showing lists of GO annotation terms significantly over- or under-represented among genes induced or repressed after exposure to peptides PAF26 and/or Melittin. Click here for file

Additional file 5 **Sensitivity of gene deletion mutants of *S. cerevisiae *to the antimicrobial peptides PAF26 and melittin and the compounds SDS and CFW**. Excel File showing the relative resistance or sensitivity to PAF26, melittin, SDS or CFW of each of the 50 gene deletion mutants assayed as compared to the reference parental strain. Click here for file

Additional file 6 **Sensitivity of *S. cerevisiae *RAY-3A and derived deletion mutants to PAF26 and Melittin**. Sensitivity assays of *S. cerevisiae *strains RAY3A and derivatives Δ*ssd1 *and Δ*pir1,2,3 *to either 32 μM Melittin or 64 μM PAF26. Click here for file

Additional file 7 **Sensitivity of *S. cerevisiae *gene deletion mutants related to MAPK pathways to peptides and SDS**. Sensitivity assays of *S. cerevisiae *gene deletion mutants related to MAPK signaling pathways, to either 32 μM Melittin, 64 μM PAF26, or 0.03% SDS. Click here for file

Additional file 8 **Oligonucleotide primers used in the quantitative RT-PCR assays**. Table showing the oligonucleotide primer sequences used for each target and reference gene to determine mRNA accumulation by quantitative RT-PCR. Click here for file
