# Supplementary material for: A genomic approach highlights common and diverse effects and determinants of susceptibility on the yeast Saccharomyces cerevisiae exposed to distinct antimicrobial peptides
Source: BMC Microbiol. 2010 Nov 15;10:289. doi: 10.1186/1471-2180-10-289 (PMC2996382; doi:10.1186/1471-2180-10-289)
Supplement: Additional file 1 — Sensitivity of S. cerevisiae strains to peptides PAF26 and Melittin. Sensitivity assays of S. cerevisiae strains RAY3A, BWG7a, FY1679, and BY4741 (105 or 104 CFU/mL) to different concentrations of peptides PAF26 and Melittin, at two different assay temperatures. [file 1471-2180-10-289-S1.PDF]

Additional File 1

Sensitivity of *S. cerevisiae* strains to peptides PAF26 and Melittin

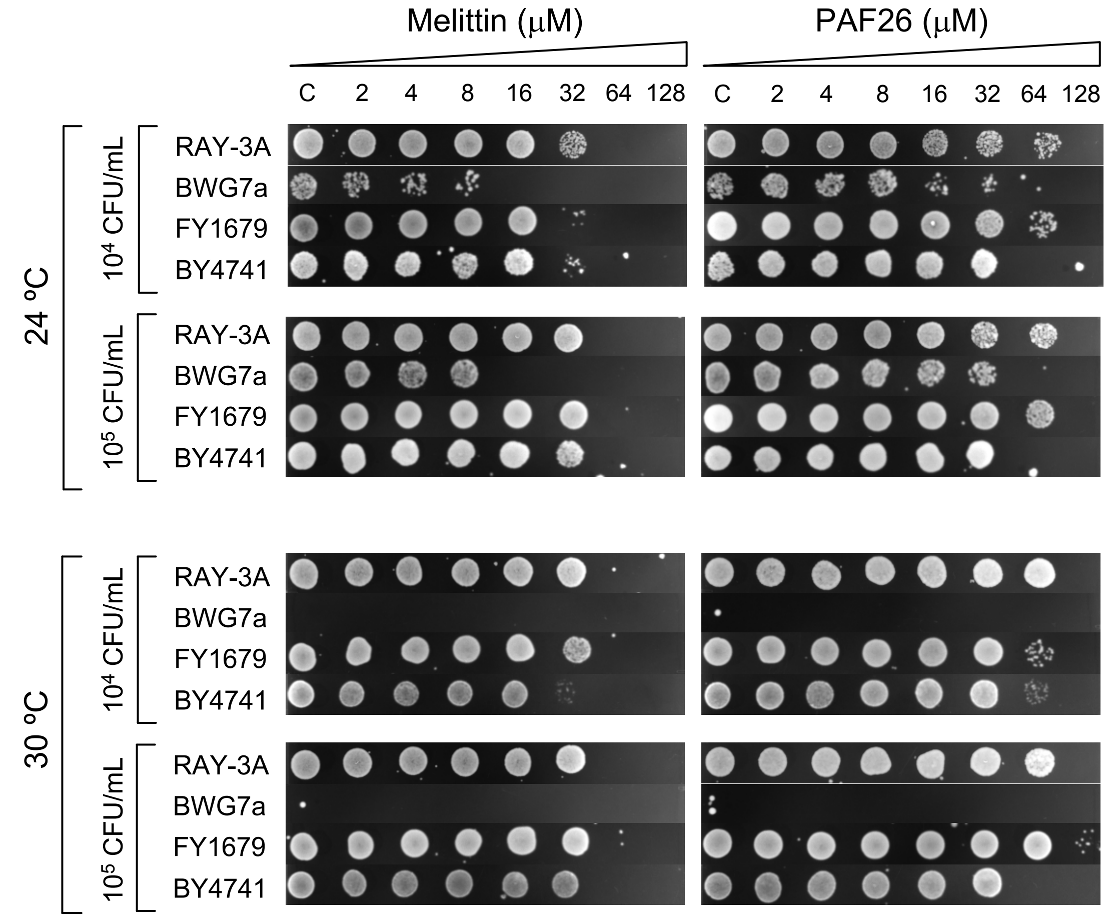

Additional File 1. Sensitivity of *S. cerevisiae* strains to peptides PAF26 and Melittin. *S. cerevisiae* strains RAY3A, BWC7a, FY1679, and BY4741 at exponential phase and diluted to two different concentrations (10^5 or 10^4 cfu/mL) were treated with different concentrations of peptides and at two distinct temperatures, as indicated. Aliquots were dotted on peptide-free YPD plates to determine viability after treatment.
