# Supplementary material for: A genomic approach highlights common and diverse effects and determinants of susceptibility on the yeast Saccharomyces cerevisiae exposed to distinct antimicrobial peptides
Source: BMC Microbiol. 2010 Nov 15;10:289. doi: 10.1186/1471-2180-10-289 (PMC2996382; doi:10.1186/1471-2180-10-289)
Supplement: Additional file 6 — Sensitivity of S. cerevisiae RAY-3A and derived deletion mutants to PAF26 and Melittin. Sensitivity assays of S. cerevisiae strains RAY3A and derivatives Δssd1 and Δpir1,2,3 to either 32 μM Melittin or 64 μM PAF26. [file 1471-2180-10-289-S6.PDF]

Additional File 6

Sensitivity of *S. cerevisiae* RAY-3A and derived deletion mutants to PAF26 and Melittin

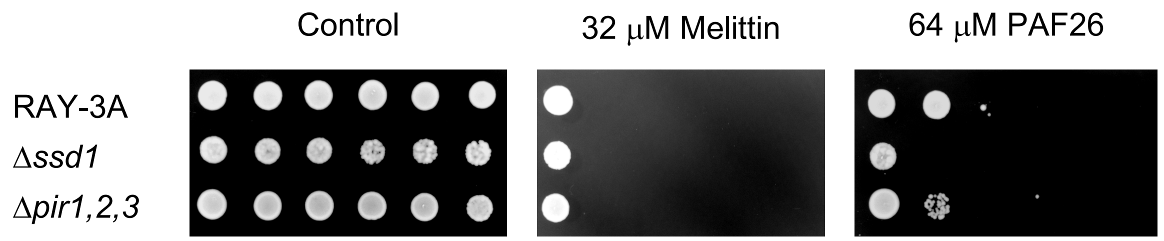

Additional File 6. Sensitivity of *S. cerevisiae* RAY-3A and derived deletion mutants to peptides PAF26 and Melittin. *S. cerevisiae* strains RAY3A and the derivatives Δ*ssd1* and Δ*pir1,2,3* (see main text for details) at exponential phase (10^7 cfu/mL) were serial 5-fold diluted and incubated with peptides at the indicated concentrations at 30ºC for 24 hours. After treatment, aliquots were applied on YPD peptide-free plates to determine viability .
