# Supplementary material for: A genomic approach highlights common and diverse effects and determinants of susceptibility on the yeast Saccharomyces cerevisiae exposed to distinct antimicrobial peptides
Source: BMC Microbiol. 2010 Nov 15;10:289. doi: 10.1186/1471-2180-10-289 (PMC2996382; doi:10.1186/1471-2180-10-289)
Supplement: Additional file 7 — Sensitivity of S. cerevisiae gene deletion mutants related to MAPK pathways to peptides and SDS. Sensitivity assays of S. cerevisiae gene deletion mutants related to MAPK signaling pathways, to either 32 μM Melittin, 64 μM PAF26, or 0.03% SDS. [file 1471-2180-10-289-S7.PDF]

Additional File 7

Sensitivity to peptides and SDS of *S. cerevisiae* deletion mutants involved in MAPK pathways

A

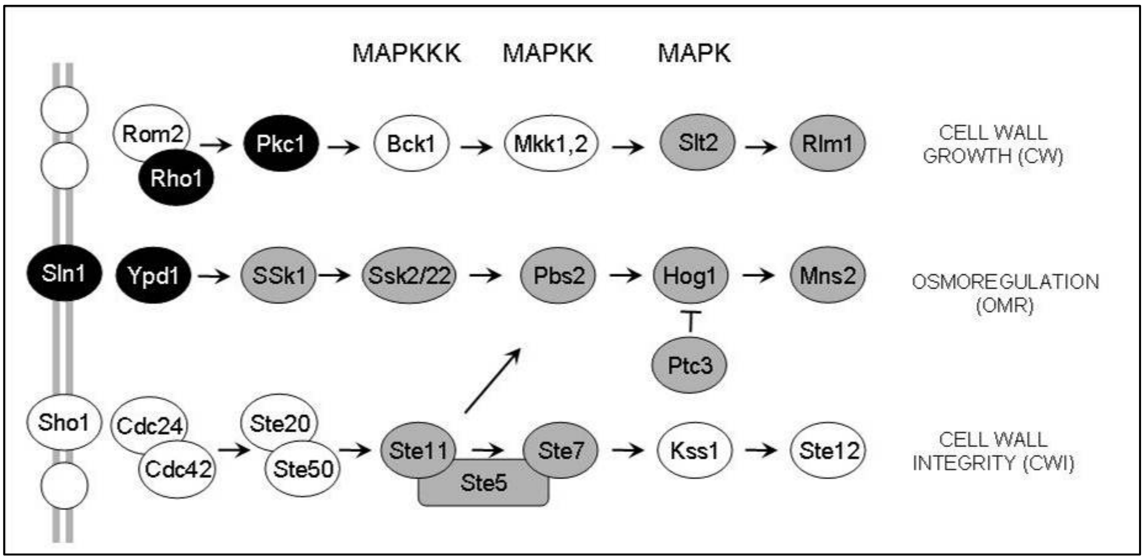

B

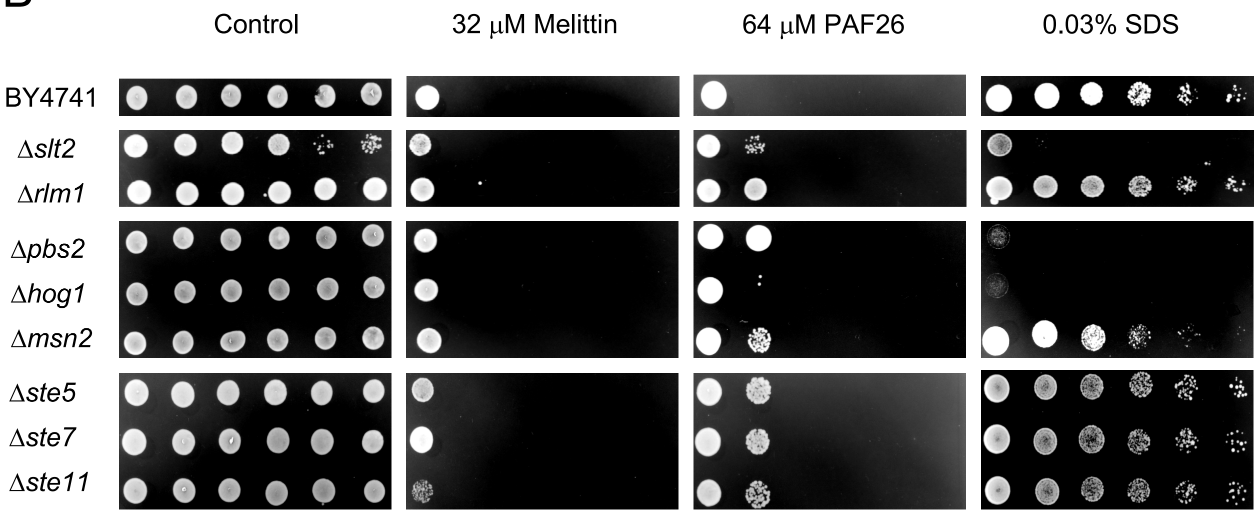

Additional File 7. Sensitivity to peptides and SDS of *S. cerevisiae* deletion mutants involved in MAPK pathways. (A) Schematic diagrams of the three CW-related MAPK signaling pathways studied (see main text for literature references). The studied haploid deletion strains appear in grey, while diploid strains appear in black. (B) Sensitivity assays of selected strains from those shown in (A). *S. cerevisiae* strains at exponential phase (10^7 cfu/mL) were serial 5-fold diluted and either applied to SDS amended YPD plates or incubated with peptides at the indicated concentrations at 30ºC for 24 hours. After peptide treatment, aliquots were applied on YPD peptide-free plates to determine viability.
