# Supplementary material for: A genomic approach highlights common and diverse effects and determinants of susceptibility on the yeast Saccharomyces cerevisiae exposed to distinct antimicrobial peptides
Source: BMC Microbiol. 2010 Nov 15;10:289. doi: 10.1186/1471-2180-10-289 (PMC2996382; doi:10.1186/1471-2180-10-289)
Supplement: Additional file 8 — Oligonucleotide primers used in the quantitative RT-PCR assays. Table showing the oligonucleotide primer sequences used for each target and reference gene to determine mRNA accumulation by quantitative RT-PCR. [file 1471-2180-10-289-S8.PDF]

Additional File 8

Oligonucleotide primer sequences used to determine mRNA accumulation of genes by quantitative Real-Time PCR

Gene	Primer	Sequence 5´- 3´
ARG1	Forward	5´-TAGACTGTATAAGGGTAACGTC-3´
	Reverse	5´-AAGAAACCGGTCAACTCATCC-3´
ARG3	Forward	5´-GACTCTTTAAAGGCCTCCACC-3´
	Reverse	5´-GTTTGGATCAGCCACAGAGAC-3´
ARG7	Forward	5´-GCTGAAGCTTGTCGCTAATGG-3´
	Reverse	5´-CGGTTCCCAAGTCGACAGAC-3´
BTN2	Forward	5´-TTGTTATTGGCTGTGGAGTTGA-3´
	Reverse	5´-TTCGTTGATGCCACCTTCGG-3´
CGR1	Forward	5´-AGGATGAGAAGGAAGAAGCTC-3´
	Reverse	5´-ATCTTAGCAGCCAAACGTTCG-3´
DSE2	Forward	5´-CTGGTACCCTCTTCTAGCGT-3´
	Reverse	5´-AACGTAACAAGTGCCATCCGA-3´
ECM33	Forward	5´-CTTCTTCTAGTGGTGATGCCT-3´
	Reverse	5´-TAGTAAGCAACGCCAACAGCA-3´
NOP16	Forward	5´-AACTTTGATGCTGATGAGGATG-3´
	Reverse	5´-TTTCCAACCATTCTTCTTCCCT-3´
PIR1	Forward	5´-AACCATTATACCATCTCCAGCT-3´
	Reverse	5´-GCAACAATAGAACCAATTCTACC-3´
PIR2	Forward	5´-CAAGCTACTACTGCTACTTCC-3´
	Reverse	5´-GCAACAATAGAACCAATTCTACC-3´
PIR3	Forward	5´-CGCTAGTCAAGTAAGTGATGG-3´
	Reverse	5´-GCAACAATAGAACCAATTCTACC-3´
PIR4	Forward	5´-AGCTCCGAAAAGATCTCTTCC-3´
	Reverse	5´-GCAACAATAGAACCAATTCTACC-3´
SED1	Forward	5´-GCTTCTTCTCATTCCGTTGTC-3´
	Reverse	5´-AACACCAGCCAAACCTAAAGC-3´
SSD1	Forward	5´-CGTTGCTGTTTTGGACCGTAT-3´
	Reverse	5´-CTTCTTATCAGTAGGCTTGAAC-3´
ACT1	Forward	5´-CCAAACTACTTACAACTCCATC-3´
	Reverse	5´-TTTCTGGAGGAGCAATGATCTT-3´
ALG9	Forward	5´-CACGGATAGTGGCTTTGGTG-3´
	Reverse	5´-GGCAGCAGGAAAGAACTTGGG-3´
TAF10	Forward	5´-CAGGATCAGGTCTTCCGTAGC-3´
	Reverse	5´-GTAGTCTTCTCATTCTGTTGATG-3´
UBC6	Forward	5´-GATACTTGGAATCCTGGCTGG-3´
	Reverse	5´-GGGTCTTCTGTTTCATCACCTG-3´
